# Adipose tissue and fat-derived products in wound, ulcer, and scar management: a systematic review

**DOI:** 10.3389/fsurg.2025.1666776

**Published:** 2025-10-09

**Authors:** Lana Sbitan, Asem Qandah, Noor Alzraikat, Cristina P. Camargo

**Affiliations:** 1 Department of General and Special Surgery, Faculty of Medicine, The Hashemite University, Zarqa, Jordan; 2 King Hussein Medical Center, Royal Medical Services, Amman, Jordan; 3 Laboratory of Microsurgery and Plastic Surgery (LIM-04), School of Medicine, Universidade de São Paulo, São Paulo, Brazil

**Keywords:** adipose tissue, fat-derived products, wound healing, ulcer treatment, scar management, regenerative medicine

## Abstract

**Introduction:**

Adipose-derived therapies hold promise in addressing the increasing prevalence of skin wounds, scars, and ulcers. This systematic review, conducted following the PRISMA guidelines, evaluates the therapeutic potential of adipose derived stem cells for improving wound healing, scar development and ulcer management.

**Methods:**

An extensive search was conducted across PubMed, EMBASE, Scopus, Web of Science, Cochrane Central, and LILACS. The search strategy employed a combination of keywords and Medical Subject Headings (MeSH) terms related to “adipose tissue”, “fat derivatives”, “ulcers”, “wound healing”, and their synonyms, covering articles published from inception to October 2024. Our search yielded 589 records, with 16 randomized clinical trials and two ongoing studies meeting inclusion criteria after screening and full-text assessment.

**Results:**

Findings indicate that adipose-derived therapies significantly enhance wound healing, reduce pain, and improve cosmetic appearance, patient satisfaction, and health-related quality of life compared to conventional treatments.

**Discussion:**

These therapies demonstrate efficacy across various wound types and scars, with a favorable safety profile. However, further standardized protocols and large-scale randomized trials are essential to validate these outcomes and assess longterm safety. While adipose-derived therapies show promise in enhancing wound healing and managing scars, ongoing research is essential to facilitate their integration into routine clinical practice.

**Systematic Review Registration:**

https://www.crd.york.ac.uk/PROSPERO/view/CRD42024503209, PROSPERO CRD42024503209.

## 1 Introduction

The prevalence of skin wounds, scars and ulcer development are increasing due to numerous factors such as aging, trauma, surgical procedures, burns, infections, and chronic diseases like diabetes, arterial insufficiency, and systemic sclerosis (1). This remains a critical medical issue and a burden on healthcare systems (2). Globally, the economic burden of chronic wounds is estimated to exceed 30 billion USD annually, driven by prolonged care, hospitalization, and complications.For example, burn wounds alone cause more than 300,000 deaths per year, according to the World Health Organization's (WHO) most recent statistics (3). Furthermore, it has been estimated that 1%–2% of the population in developed countries will experience a chronic wound in their lifetime (4). In the United States, documented data demonstrate that approximately six million individuals suffer from persistent non-healing wounds, leading to an increase in healthcare costs of up to 25 billion USD (2, 5).

In many cases, wounds and scars have severe long-term consequences for patients and can significantly affect their quality of life, beyond just their cosmetic impact (6). Usually, the longer a wound takes to heal, the higher the risk of critical complications, such as amputation, organ loss or even death (7). The alarming statistic that 70% of amputations result from persistent non-healing wounds highlights the urgency to address this prevalent issue and explore innovative techniques to reduce suffering and the economic burden caused by wounds (8).

Thus, emerging interest in the role of regenerative medicine and tissue engineering in wound healing has been a hot topic in modern research. The use of several types of stem cells, such as mesenchymal stem cells (MSCs) and adipose-derived stem cells (ADSCs), for improving wound healing, repair, and preventing scar development has been investigated, particularly focusing on stem cells' regenerative effects on fibroblast proliferation through paracrine signaling and the release of growth factors (9, 10).

Special interest in human adipose tissue derivatives as potent native biomaterials for tissue regenerative therapies has been explored. The wide availability, simple processing, unique continuous remodeling ability, and richness in biomaterials that mimic the native tissue microenvironment have all contributed to research aimed at investigating the role of adipose tissue and fat derivatives in wound healing, scar development, and ulcer management (10, 11). Key derivatives of human liposuctioned adipose tissue includes, fat grafts (FG), adipose-derived stem cells (ADSCs), adipose-derived stromal vascular fraction (ADSVF) and extracellular matrix (ECM), which can be engineered alone or used with polymers for regenerative medicine and tissue engineering (11).

Although fat grafting is a popular method for addressing volume and contour irregularities in aesthetic and reconstructive surgery (12). Fat tissue contains mature adipocytes, preadipocytes, stem cells, and growth factors, with a lower risk of triggering an immune response, and it plays a significant role in regeneration and remodeling (11). ADSCs represent a group of MSCs that can be obtained easily from adipose tissue and have similar regenerative properties as other MSCs (13). Additionally, ADSVF is a derivative of adipose tissue that contains heterogeneous cell populations such as mesenchymal progenitor/stem cells, preadipocytes, endothelial cells, pericytes, T cells, and M2 macrophages (14). Furthermore, adipose-derived stem cell-conditioned media (ADSC-CM) contains cytokines and growth factors that play a role in facilitating the tissue repair process (15).

Given the challenges and limitations surrounding wound healing treatments' efficacy and complications, this systematic review aims to evaluate the clinical efficacy and applications of adjunctive use of adipose tissue and fat derivatives in the treatment of skin wounds, scars, and ulcers. Through synthesizing existing evidence, this review focuses on investigating the gaps in current knowledge and explores the implications of adipose-derived therapies in improving clinical outcomes.

## 2 Materials and methods

This systematic review was conducted and described according to the Preferred Reporting Items for Systematic reviews and Meta-Analyses (PRISMA) statement (16). A protocol was developed beforehand and registered in PROSPERO (ID: CRD42024503209).

### 2.1 Data sources and search strategy

We searched the following databases: PubMed, EMBASE, Scopus, Web of Science, Cochrane Central Register of Controlled Trials, and Latin American & Caribbean Health Sciences Literature (LILACS) from inception to October 2024, using a well-developed search strategy that included terms such as “ulcer”, “adipose stem cell”, and “wound”, along with their synonyms. We conducted a manual search of references in eligible studies and ongoing clinical trials listed in trials registries (ClinicalTrials.gov) and conference proceedings. The complete search strategy can be found in the Appendix 1.

### 2.2 Study selection

During preliminary screening, two independent authors (L.S. and N.A.) reviewed articles for inclusion based on the title, abstract, and methods of each article. Full-text articles were retrieved in the second round of screening, and articles were included based on the following eligibility criteria: clinical trials involving (I) individuals with any form of wounds or scars subjected to various fat derivative treatments and (II) participants of all genders and age groups. Studies involving animals, laboratory experiments, *in vitro* research, conference presentations, reviews, and book chapters; as well as study designs such as case reports, case series, and cross-sectional studies; and non-English studies were excluded. Any discrepancies were resolved through discussion with a third reviewer (C.P.C).

### 2.3 Data extraction and synthesis

A uniform data extraction form was developed, and two independent authors (L.S. and N.A.) extracted pertinent data from each eligible study. The extracted information included the following domains: reference (first author and year of publication), study design, sample size, demographic characteristics (i.e., age and gender), intervention, control, success of the intervention (wound healing), and observed adverse events post-intervention. Given the heterogeneous nature of the studies included, conducting a meta-analysis was not feasible. As a result, a qualitative synthesis approach was adopted, and these studies' findings were compared descriptively.

### 2.4 Quality assessment

We used the Cochrane risk-of-bias tool for randomized trials (RoB v2) to conduct a quality assessment of the included studies (17).We evaluated the following domains: the randomization process, deviations from intended interventions, missing outcome data, outcome measurements, and selection of the reported results.

## 3 Results

### 3.1 Identiﬁcation and selection of studies

**Figure 1:**
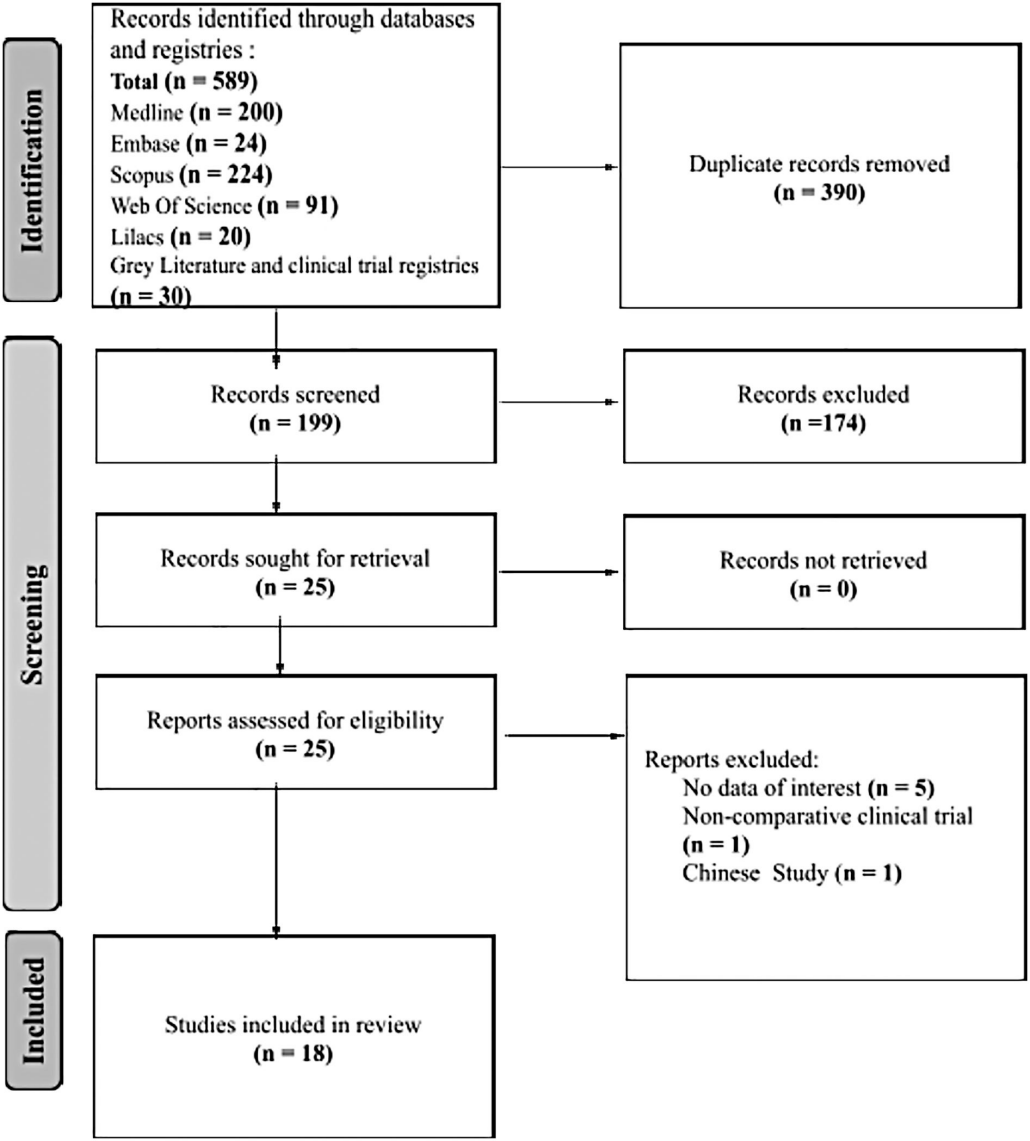
A flow chart outlining the systematic process of study identification and selection for a review. It begins with an initial database search, followed by screening for relevance, full-text assessment, and inclusion of selected studies in the systematic review.

The database search yielded 589 records. After removing duplicates, 199 studies underwent an initial screening based on titles and abstracts. Of these, 174 did not meet our inclusion criteria. Full-text copies of 23 studies were then obtained for further evaluation, and two ongoing trials were assessed using published data on clinical registries. Through a collaborative review by all authors, 7 studies were excluded, leaving 18 studies that met the inclusion criteria for this systematic review (refer to Figure 1) (17–34).

### 3.2 Quality assessment

The overall assessment of the risk of bias for the 16 randomized clinical trials included in this review, conducted using the Cochrane risk-of-bias tool (ROB v2), yielded the following results:

- Eight studies were assessed as having a low risk of bias (17, 20, 26, 27, 29–32).
- Three studies had an unclear risk of bias (some concerns) (18, 22, 23).
- Five studies were categorized as having a high risk of bias (19, 21, 24, 25, 28).

In reference to the randomization domain, all included studies had a low risk of bias in that area (17–32). Assessing the deviation from the intended interventions domain, 4 studies had a high risk of bias (19, 21, 25, 28), and 4 studies had an unclear risk (18, 22–24). Concerning missing outcome data, only one study exhibited a high risk of bias (24). In the domain of outcome measurement, 2 studies had some concerns in that area (18, 24), while one study (19), was classified as having a high risk of bias.

In terms of selection bias, all the included studies exhibited a low risk (see Table 1) (17–32).

**Table 1:** Quality assessment of included clinical trials using (RoB V2) quality assessment tool.

Author, year	Randomization	Deviations from intended interventions	Missing outcome data	Outcome measurement	Selection of the reported results	Overall bias
Cervelli et al. (2012) (17)	Low	Low	Low	Low	Low	Low-Risk
Zhou et al. (2013) (18)	Low	Unclear	Low	Unclear	Low	Unclear Risk
Zollino et al. (2019) (19)	Low	High	Low	High	Low	High Risk
Del Papa et al. (2019) (20)	Low	Low	Low	Low	Low	Low Risk
Smith et al. (2020) (21)	Low	High	Low	Low	Low	High Risk
Nolan et al. (2022) (25)						
Kemaloğlu et al. (2021) (22)	Low	Unclear	Low	Low	Low	Unclear Risk
Tanios et al. (2021) (23)	Low	Unclear	Low	Low	Low	Unclear Risk
Van Dongen et al. (2022) (24)	Low	Unclear	High	Unclear	Low	High Risk
Roohaninasab et al. (2022) (26)	Low	Low	Low	Low	Low	Low Risk
Behrangi et al. (2022) (27)	Low	Low	Low	Low	Low	Low Risk
Abouzaid et al. (2022) (28)	Low	High	Low	Low	Low	High Risk
Kwon et al. (2023) (29)	Low	Low	Low	Low	Low	Low Risk
Iglesias et al. (2023) (30)	Low	Low	Low	Low	Low	Low Risk
Thamm et al. (2023) (31)	Low	Low	Low	Low	Low	Low Risk
Alinda et al. (2023) (32)	Low	Low	Low	Low	Low	Low Risk

### 3.3 Ongoing clinical trials

By searching clinical trial registries, two ongoing randomized clinical trials were identified. The first study explores the use of fat grafting in treating Post-Mastectomy Radiation Injury in breast cancer patients (33), while the other aims to assess the efficacy of microsized adipose tissue in treating diabetic foot ulcers (Refer to Table 2) (34).

**Table 2:** Overview of ongoing clinical trials examining various forms of fat-derived treatments for wounds.

Title	Country	Study design	Registration date	Intervention	Control
Adipose-Induced Regeneration of Breast Skin to Treat Post-Mastectomy Radiation Injury in Breast Cancer Patients (33)	United States	Randomized, Prospective Pilot Study	6/7/2019	Fat grafting (patients will receive their fat grafting procedure until 6 months after their second stage breast reconstruction)	Fat grafting (patients will receive fat grafting procedure per standard of care during their Second stage breast reconstruction)
Multi-Center, Randomized Controlled Clinical Investigation Evaluating the Effect of Adipose Tissue Processed With the SyntrFuge™ System in the Healing of Diabetic Foot Ulcers (34)	United States	Randomized clinical trial	8/25/2022	Microsized adipose tissue	Standard of Care (Offloading)

### 3.4 Characteristics of included studies and publication trends

Table 3 provides an overview of the demographic characteristics of patients across all clinical trials. Our review included 537 patients, excluding those lost to follow-up, with an age range spanning from 15 to 78 years. The studies included a wide variety of wound and ulcer types: six studies focused on the effects of adipose tissue treatments on chronic lower leg ulcers (19, 21, 23, 25, 31, 32), while four examined post-surgical and traumatic scars (17, 22, 24, 29), including two that specifically addressed post-reduction mammoplasty scars (22, 24). Additionally, two trials explored treatments for acne scars (26, 27), and one study delved into the role of adipose tissue and its derivatives in post-laser resurfacing scars (18). Furthermore, two clinical trials centered on patients with ischemic digital ulcers due to systemic sclerosis (20, 30), and one trial focused on patients with superficial and deep dermal burn wounds (28).

**Table 3:** Characteristics of included studies and patients.

Author, year	Country	Patients characteristics								
		Sample size			Age (years)			Gender [Males(M) | Females (F)]		
		Total	Intervention	Control	Total	Intervention	Control	Total	Intervention	Control
Cervelli et al. (2012) (17)	Italy	60	Group A (20) Group C (20)	Group B (20)	(Mean ± SD) (38 ± 16) (Range) (22–54)	(Mean ± SD) Group A (37 ± 16) Group C (38 ± 15)	(Mean ± SD) Group B (38 ± 16)	30M|30F	Group A (10M|10F) Group C (9M |11F)	Group B (11M|9F)
Zhou et al. (2013) (18)	China	19	19 (Self-controlled—bilateral inner arms)		Range (24-33)	Range (24-33)		5M|14F	5M|14F	
Zollino et al. (2019) (19)	Italy	16	8	8	NA	(Mean ± SD) 74 ± 6.7	(Mean ± SD) 68 ± 12.8	10M|6F	5M|3F	5M|3F
Del Papa et al. (2019) (20)	Italy	38	25	13	Range (21-70)	(Median + Range) 42 (21–69)	(Median + Range) 37 (23–70)	2M|36F	2M|23F	0M|13F
Smith et al. (2020) (21)	UK (Two papers reporting the results of the same clinical trial)	18	Group 1 (6) Group 2 (6)	Group 3 (6)	Mean(Range) 57.6 (35-78)	Mean(Range) Group 1 60.2 (45-78) Group 2 57.5 (35–71)	Mean(Range) Group 3 55.2 (41–69)	15M|3F	Group 1 (6M|0F) Group 2 (5M|1F)	Group 3 (4M|2F)
Nolan et al. (2022) (25)										
Kemaloğlu et al. (2021) (22)	Turkey	45	Group 1 (15) Group 2 (15)	Group 3 (15)	NA	(Mean ± SD) Group 1 (37.1 ± 12.4) Group 2 (37.4 ± 11.2)	(Mean ± SD) Group 3 (35.3 ± 10.5)	NA	NA	NA
Tanios et al. (2021) (23)	Egypt	100 (95^a^)	50 (47^a^)	50 (48^a^)	Range (15–70)	(Mean ± SD) 48.12 ± 14.58	(Mean ± SD) 48.04 ± 10.56	53M|47F	29M|21F	24M|26F
Van Dongen et al. (2022) (24)	The Netherlands	40 (31^a^)	40 (Self-controlled—bilateral wise pattern reduction mammoplasty)		(Mean ± SD) 40 ± 13 Range (18–58)	(Mean ± SD) 40 ± 13 Range (18–58)		0M|40F	0M|40F	
Roohaninasab et al. (2022) (26)	Iran	10	10 (Self-controlled)		(Mean ± SD) 35 ± 4	(Mean ± SD) 35 ± 4		5M|5F	5M|5F	
Behrangi et al. (2022) (27)	Iran	10 (7^a^)	10 (Self-controlled)		NA	NA	NA	NA	NA	NA
Abouzaid et al. (2022) (28)	Egypt	100	50	50	NA	(Mean ± SD) 26.2 ± 9.6	(Mean ± SD) 29 ± 8.6	58M|42F	30M|20F	28M|22F
Kwon et al. (2023) (29)	South Korea	20 (16^a^)	20 (16^a^) (Self-controlled)		(Mean ± SD) 35.62 ± 13.19	(Mean ± SD) 35.62 ± 13.19		6M|10F	6M|10F	
Iglesias et al. (2023) (30)	Mexico	20 19^a^)	10	10 (9^a^)	NA	(Mean,95%CI) 55.0 (43.4,58.7)	(Mean,95%CI) 57.0 (45.6,62.5)	1M|19F	0M|10F	1M|9F
Thamm et al. (2023) (31)	Germany	34 (31^a^)	17	17 (14^a^)	(Mean ± SD) 61 ± 13	(Mean ± SD) 62 ± 11	(Mean ± SD) 60 ± 14	26M|5F	13M|4F	13M|1F
Alinda et al. (2023) (32)	Indonesia	32	16	16	Mean ± SD 45.47 ± 6.10 Range (32–54)	Mean ± SD 45.75 ± 4.97 Range (38–52)	Mean ± SD 45.19 ± 7.22 Range (32–54)	19M|13F	10M|6F	9M|7F

NA, not available.

a Excluding patients lost to follow-up.

The first randomized clinical trial we included was published in 2012 (17). The largest number of studies, totaling nine, was published in the years 2022–2023 (24–32). Among these, three studies were conducted in Italy (17, 19, 20), two in the UK (21, 25), two in Egypt (23, 28), and two in Iran. The remaining studies were conducted in China (18), Turkey (22), the Netherlands (24), South Korea (29), Mexico (30), Germany (31), and Indonesia (32).

### 3.5 Adipose derived stem cells-conditioned media (ADSC-CM)

Our review included two RCTs investigating the use of ADSC-CM in wound healing (18, 32). Zhou et al. recruited patients with scars following anti-photoaging therapy—ablative fractional carbon dioxide laser resurfacing (FxCR)—and compared adipose-derived stem cell-conditioned medium (ADSC-CM) to Dulbecco's Modified Eagle Medium (DMEM) without fetal bovine serum (FBS). Alinda et al. investigated the effect of using topical ADSC-CM on treating chronic plantar ulcers after Leprosy, comparing it to Framycetin gauze dressing (Refer to Table 4).

**Table 4:** Overview of studies investigating the use of adipose tissue and fat-derived products in wound, ulcer, and scar management.

Author, year	Wound/ulcer/scar	Intervention	Control
Adipose-derived stem cells-conditioned media (ADSC-CM)			
Zhou et al. (2013) (18)	Ablative Fractional carbon dioxide laser resurfacing (FxCR) Scars	Allogeneic adipose-derived stem cells-conditioned media	Fetal bovine serum (FBS) free Dulbecco's modified Eagle's medium (DMEM)
Alinda et al. (2023) (32)	Chronic Plantar Ulcers after Leprosy	Adipose mesenchymal stem cell-conditioned medium (every three days for eight weeks)	Framycetin gauze dressing(every three days for eight weeks)
Adipose derived stromal vascular fraction (ADSVF)			
Zollino et al. (2019) (19)	Chronic leg ulcers	Centrifuged adipose tissue derived stromal vascular fraction	No experimental treatment was given to the control group
Tanios et al. (2021) (23)	Chronic ulcers (diabetic, venous, trophic and post-traumatic)	Adipose derived stromal vascular fraction	Conventional treatment (wound dressings)
Van Dongen et al. (2022) (24)	Bilateral reduction mammoplasty scars	Tissue stromal vascular fraction of adipose tissue	Saline Injection (Placebo)
Kwon et al. (2023) (29)	Traumatic and surgical scars	Stromal vascular fraction injection	Normal saline Injection (Placebo)
Roohaninasab et al. (2022) (26)	Acne scars	Subcision technique with stromal vascular fraction injection	Subcision technique only
Behrangi et al. (2022) (27)	Acne scars	Combination of nanofat subcutaneously and Stromal vascular Fraction intradermally	Nanofat subcutaneously
Iglesias et al. (2023) (30)	Systemic sclerosis wounds (digital ulcers)	Medical treatment and Local adipose derived stromal vascular fraction mixed with micrografts	Medical treatment only (stable vasoactive and immunosuppressive therapies)
Fat grafting with platelet rich plasma (FG + PRP)			
Cervelli et al. (2012) (17)	Traumatic scars	- -Group A (fat grafts mixed with PRP during months 1 and 3). - Group C (Graft⁄PRP treatment, with the laser therapy during months 1 and 3 delivered 7 days after the graft⁄PRP treatment)	- Group B [four sessions of laser treatment with the 1,540 nm non ablative laser (one per month)]
Smith et al. (2020) (21)	Diabetic foot ulcers (Two papers reporting the results of the same clinical trial)	- Group 1 (fat grafting) - Group 2 (fat grafting with PRP)	- Group 3 (podiatry standard of care)
Nolan et al. (2022) (25)			
Fat grafting without platelet rich plasma			
Del Papa et al. (2019) (20)	Digital Ischemic ulcers (IDU) in patients with systemic sclerosis	Fat grafting with medical treatment	Sham Procedure (false liposuction and local injection of saline solution) with medical treatment
Kemaloğlu et al. (2021) (22)	Post-Reduction mammoplasty scars	- Group 1 (Fat Graft) - Group 2 (Nanofat-enriched fat graft)	- Group 3 (No additional treatment was applied to the surgical incisions)
Abouzaid et al. (2022) (28)	Superficial and deep dermal burn wounds	Single injection of autologous fat grafting and dressing with nanofat	Conventional methods with serial dressing and use of topical agents e.g., Silver Sulfadiazine, mafenide and/or others
Thamm et al. (2023) (31)	Chronic leg ulcers	Sublesional fat graft Injection	Saline solution (0.9% NaCl) injection

#### 3.5.1 Intervention success: wound healing

In Zhou et al. (18) study, The ADSC-CM treated side presented statistically significantly lower erythema and hyperpigmentation levels compared to the control side [as assessed by the erythema index (EI) and the melanin index (MI), respectively]. On the other hand, a similar reduction in trans-epidermal water loss (TEWL) level was detected in both groups. No differences in histopathologic examination were recorded.

Compared to 10 (62.5%) patients in the intervention group in the Alinda et al. trial, only 4 (25.0%) patients in the control group had full healing (32). Additionally, the number of patients reporting improved healing in ADSC-CM and Framycetin groups were 6 patients and 12 patients, respectively.

Furthermore, statistically significant reductions in ulcer size and depth, as well as higher vascularity value were assessed in the ADSC-CM group (*P* value <0.05).

##### 3.5.1.1 Post-intervention adverse events

Adverse events and complications including (infection, prolonged erythema, scarring, and allergic contact dermatitis) were not observed in either the intervention or the control groups in both trials (see Supplementary Table 1) (18, 32).

### 3.6 Adipose derived stromal vascular fraction (ADSVF)

Seven RCTs included in our review investigated the use of stromal vascular fraction in the management and healing of wounds, scars, and ulcers (19, 23, 24, 26, 27, 29, 30).

#### 3.6.1 Chronic ulcers

Both Zollino et al. and Tanios et al. focused on the use of ADSVF in the management of chronic ulcers, including diabetic, venous, trophic, and post-traumatic ulcers (19, 23). Zollino et al. compared ADSVF to no experimental treatment (19), whereas Tanios et al. used conventional treatment (wound dressings) in their control group (Table 4) (23).

##### 3.6.1.1 Intervention success: wound healing

In Zollino et al.'s study, statistically significant differences were reported for the ADSVF group in terms of mean healing time and pain score (*P* = 0.036, *P* < 0.01, respectively) (19). However, the healing rate at 24 weeks, wound healing process, and the Margolis Index showed no statistically significant differences between the experimental and control groups (*P* = 0.30, *P* = 0.37, and *P* = 0.30, respectively) (19).

Tanios et al. reported a statistically significant higher complete healing rate at 9 weeks in the ADSVF group (46 out of 47 patients who completed the follow-up period) compared to the control group (30 out of 48 patients) (*P* < 0.001). Additionally, the intervention group demonstrated a significantly shorter healing duration (*P* = 0.000) (refer to Supplementary Table 2) (23).

##### 3.6.1.2 Post-intervention adverse events

In terms of adverse events, Zollino et al. reported one case of perilesional dermatitis in the ADSVF group (19). In the trial by Tanios et al., three patients in the ADSVF group experienced post-intervention infection (23). However, when compared to the rate of post-intervention infection in the control group (*n* = 14), it was significantly lower (*P* = 0.000) (23).

#### 3.6.2 Post-surgical and traumatic scars

Van Dongen et al. specifically explored the role of using tissue stromal vascular fraction of adipose tissue (tSVF) in the treatment of post-reduction mammoplasty scars, comparing it to saline injection (placebo) (24). Meanwhile, Kwon et al. investigated the effect of stromal vascular fraction (SVF) on traumatic and post-surgical scars in general, also comparing it to the use of normal saline injection (29).

##### 3.6.2.1 Intervention success: wound healing

Both studies utilized the Patient and Observer Scar Assessment Scale (POSAS) to evaluate post-intervention scar appearance (24, 29). They both reported significant improvements in scar appearance at the 6-month follow-up (*P* < 0.05) (24, 29). However, Van Dongen et al. noted that this significant improvement was not sustained at the 12-month follow-up (*P* > 0.05) (24). Additionally, they reported no effect of tSVF on collagen architecture enhancement (see Supplementary Table 2) (24).

##### 3.6.2.2 Post-intervention adverse events

While the study by Van Dongen et al. did not report adverse events post-intervention (24), Kwon et al. investigated the occurrence of various complications among the patients, including bleeding, infection, fat necrosis, skin necrosis, systemic allergic or anaphylactic reactions, fever, headache, muscle pain, or fatigue (29). Only two patients reported mild pain two days following SVF injection (29).

#### 3.6.3 Acne scars

We included two clinical trials focusing on the use of SVF to treat acne scars. Roohaninasab et al. compared the use of the Subcision technique with SVF injection in the intervention group to the Subcision technique only (26). Meanwhile, Behrangi et al. compared the combination of nanofat subcutaneously and SVF intradermally to nanofat subcutaneously alone (27).

##### 3.6.3.1 Intervention success: wound healing

Both studies reported significant improvements in scar variables such as volume and area in the SVF group (*P* < 0.05) (26, 27). However, only Behrangi et al.'s study showed a significant improvement in scar depth (*P* < 0.05) (27), while Roohaninasab et al. did not report a significant improvement (*P* = 0.438) (26).

Regarding sonographic scar measurements, including epidermal and dermal thickness, as well as epidermal density variables, Roohaninasab et al. reported significant improvement (*P* < 0.05) (26), whereas Behrangi et al. reported insignificant differences (*P* > 0.05) (27).

Concerning patient and doctor satisfaction with scar appearance, Roohaninasab et al. demonstrated significant differences in the SVF group (*P* = 0.003, *P* = 0.004, respectively) (refer to Supplementary Table 2 for more details) (26).

##### 3.6.3.2 Post-intervention adverse events

No incidence of bleeding or infection at the sites of fat removal and Subcision were observed in Roohaninasab et al.'s study (26), whereas Behrangi et al. did not investigate post-intervention adverse events (27).

#### 3.6.4 Systemic sclerosis ulcers

In our review, Iglesias et al. was the only study investigating the effect of using ADSVF with micrografts in the management of digital ulcers in patients with systemic sclerosis, in addition to the medical treatment administered to patients, and comparing it to medical treatment only (30).

##### 3.6.4.1 Intervention success: wound healing

In terms of the number of digital ulcers, Raynaud Phenomenon, quality of life, and pain assessment, the ADSVF group reported significant differences compared to the control (*P* < 0.05) (30). However, no differences were reported in digital oximetry (SpO2), digital total active motion, thumb opposition, hand function, health status and disability index, and nail capillaroscopic pattern (*P* > 0.05)(see Supplementary Table 2) (30).

##### 3.6.4.2 Post-intervention adverse events

Ulcer recurrence was reported in both the intervention and control groups (*n* = 1 and *n* = 3, respectively) (30).

### 3.7 Fat grafting with platelet-rich plasma (FG + PRP)

We included three studies investigating the effect of using Fat Grafting with Platelet-Rich Plasma (FG + PRP) in the management of scars and ulcers (17, 21, 25). Cervelli et al. explored the effect of (FG + PRP) on traumatic scars by dividing the patients into three groups: two intervention groups receiving (FG + PRP) alone and (FG + PRP) with non-ablative resurfacing laser therapy, respectively, while the control group received non-ablative resurfacing laser therapy alone (17). Smith et al. and Nolan et al. studies, two papers reporting the results of the same clinical trial, investigated the role of (FG + PRP) in the healing of diabetic foot ulcers (21, 25). The trial included three arms: FG alone, (FG + PRP), both compared with standard podiatry care as the control (Table 4) (21, 25).

#### 3.7.1 Intervention success: wound healing

Cervelli et al. reported a significant difference in the effectiveness of scar treatments with the addition of PRP, emphasizing that the most effective scar treatment was the combination of (FG + PRP) and non-ablative laser resurfacing (17). Regarding patient satisfaction, the majority of patients were satisfied with the treatment, with no significant differences reported between groups (17).

The second clinical trial did not report any significant differences between the intervention and control groups in terms of wound size, healing, or Pressure Ulcer Scale for Healing (PUSH score) (21, 25). However, significant improvements in health-related quality of life were observed in both intervention groups (refer to Supplementary Table 3 for more details) (21, 25).

#### 3.7.2 Post-intervention adverse events

Both clinical trials reported adverse events post-intervention generally, without specifying which group experienced them (17, 21, 25).

### 3.8 Fat grafting without platelet-rich plasma

In our systematic review, we included four clinical trials investigating the use of fat graft injection in the wound healing process (20, 22, 28, 31). Del Papa et al. compared the effects of fat injection to normal saline injection on the healing of ischemic digital ulcers in systemic sclerosis patients, while Thamm et al. studied the effects of the same intervention and control on chronic ulcer wounds, including venous, arterial, mixed arterial–venous, diabetogenic, and compressive ulcers (20, 31). Both Kemaloğlu et al. and Abouzaid et al. examined the effects of using nanofat (22, 28). Kemaloğlu et al. divided post-reduction mammoplasty patients into three groups: the first intervention group received fat injection, the second group received nanofat injection, while the control group did not receive any additional post-surgical treatment for scars (22). On the other hand, Abouzaid et al. combined both fat injection and nanofat dressing as interventions in patients with superficial and deep dermal burn wounds, comparing them to conventional dressing (Table 4) (28).

#### 3.8.1 Intervention success: wound healing

Del Papa et al. reported a statistically significant higher rate of complete healing, number of capillaries, and pain improvement in the fat graft group compared to the control (*P* < 0.0001) (20). However, although Thamm et al. observed better wound parameters in the experimental group, the results regarding wound size, neovascularization, and pain levels were not statistically significant (*p* > 0.05) (31).

Kemaloğlu et al. documented significantly better results in post-mammoplasty scars and pain levels in both the Fat and Nanofat groups compared to the control group. However, when comparing the Fat and Nanofat groups, only the Nanofat group reported statistically significant improvements in pigmentation scores (*p* = 0.005), with no other discernible differences (22).

Combining fat graft injection followed by nanofat dressing in the management of burn wounds resulted in statistically significant differences in healing time, contracture formation, scar texture, and pain levels compared to the conventional method of burn wound management (*p* < 0.05), as reported by Abouzaid et al. (Refer to Supplementary Table 3 for further details) (28).

#### 3.8.2 Post-intervention adverse events

Local wound infection was reported in three patients in Thamm et al.'s study (31), in contrast to Del Papa et al., who reported no adverse events observed post-intervention (20). While Abouzaid et al. did not report adverse events (28), Kemaloğlu et al. reported one case of nipple necrosis in the control group (22).

## 4 Discussion

### 4.1 Summary of findings

Wound persistence significantly impacts individuals' quality of life and increases morbidity and mortality rates. Regenerative medicine is a promising approach, offering opportunities to accelerate and enhance the process of wound healing. The evolving field of regenerative medicine coupled with personalized medicine could foster the advancement of wound healing therapies (35).

Our systematic review included 16 randomized clinical trials and two ongoing studies that evaluated safety and efficacy of adipose tissue and fat derivatives for various wounds in 537 patients. Different types of adipose tissue and fat derivatives were used across the studies; seven studies used adipose-derived stromal vascular fraction (19, 23, 24, 26, 27, 29, 30), two studies used adipose derived stem cells-conditioned media (18, 32), two studies used fat grafting (20, 22, 28, 31), and two studies used fat grafting with platelet rich plasma (17, 21, 25).

Our findings demonstrated that adipose tissue and fat derivatives significantly enhance wound healing parameters, reduce pain, and improve cosmetic appearance, patient satisfaction and health-related quality of life compared to conventional treatments.

The majority of studies reported no serious adverse events related to intervention, indicating a favorable safety profile. Mild and transient adverse events like perilesional dermatitis, post-intervention local infection, and mild localized pain were reported in twelve patients across different interventions (19, 21, 23, 25, 29, 31).

It is critical to note that multiple “positive” outcomes reported were derived from clinical trials that we labelled as having high or some concerns risk of bias via our risk assessment, especially in the domains of deviation from the intended interventions, missing outcome data, and outcome measurement domains. This highlights the possibility that observed benefits may be overestimated. Thus, the strength of evidence surrounding adipose-derived therapies promising potential remains limited due to methodological weaknesses, caution should be taken while interrupting these findings.

### 4.2 Adipose derived stem cells-conditioned media

Easier accessibility from subcutaneous liposuction in large numbers and the absence of ethical and political issues concerning the collection process make adipose-derived stem cells superior over other stem cells (36, 37). ADSCs are collected, isolated and then cultured mostly under standard conditions. Conventionally, this involves Dulbecco's modified Eagle medium (DMEM) with 10% fetal bovine serum (FBS) and 1% antibiotics, maintained under 37°C and 5% CO2 in monolayer dishes (37). After 48–72 h of *in vitro* culture, ADSC-CM is collected via centrifugation (38).

Two mechanisms of action have been proposed to explain the role of ADSCs in wound healing, mainly by direct differentiation into skin cells and through paracrine secretion of growth factors, immune factors, chemokines and exosomes (39). Other studies have demonstrated the role of ADSC-CM in stimulating the migration of dermal fibroblasts and keratinocytes after wounds, reducing the wound size and accelerating the reepithelialization process at the wound edges (40–42).

Our review highlighted the positive effects of using ADSC-CM in treating superficial scars and chronic ulcers by reducing erythema, hyperpigmentation, and ulcer size and depth (18, 32). Our findings agree with Heydari et al., which investigated the efficacy of Mesenchymal stromal cell (MSC)-conditioned medium (CM) on skin wound healing *in vitro* models (43). Their findings showed the efficacy of all types of MSC-CM, including ADSC-CM, in promoting wound healing (43). This alignment suggests that ADSC-CM's positive results are not only observed *in vitro* but also *in vivo*, which helps guide further clinical studies for personalized use of ADSC-CM in skin wounds, especially chronic ulcers.

Despite the lack of actual adipose stem cells, limiting the potential of direct cell replacement, and the challenges due to the variability in preparation compositions, ADSC-CM has multiple advantages in clinical practice. ADSC-CM can be directly applied to the skin without causing immune reactions, unlike allogeneic ADSCs, reflecting the safety profile of ADSC-CM (18). Additionally, the feasibility of mass manufacturing using well-monitored laboratory systems and storage without the use of toxic cryopreservatives are further advantages (44).

### 4.3 Adipose derived stromal vascular fraction

ADSVF is composed of smooth muscle cells, fibroblasts, endothelial cells, pericytes and ADSCs acquired through washing, enzymatic digestion, filtration, and centrifugation of adipose tissue (11, 45). Although the proposed mechanism of action is similar to ADSCs, the presence of additional cellular components in ADSVF augments the mechanical framework of repaired tissues and help in the regeneration of damaged tissues by secreting cytokines (46, 47).

Our review covered seven clinical trials investigating ADSVF's role in skin wound healing across various conditions including chronic ulcers, post-traumatic and surgical scars, acne scars, and systemic sclerosis digital ulcers (19, 23, 24, 26, 27, 29, 30). The overarching finding is that ADSVF consistently promotes significant improvements in wound healing outcomes regardless of the clinical assessment parameters used.

In scar management, our findings are consistent with Mbiine et al. and Stachura et al., where they investigated the role of ADSVF in the management of hypertrophic, keloid, and scar treatment (48, 49). Both studies demonstrated the efficacy of ADSVF in improving scar appearance, similar to our findings (48, 49). Similarly, studies on ADSVF in breast surgery were also consistent with our findings demonstrating better outcomes and increased patient satisfaction (50, 51). However, Li and Chen (2021) found no significant differences between ADSVF and conventional fat grafting, especially in terms of fat survival rate, mainly due to the heterogeneity of methods used for ADSVF extraction in the included studies (52).

A recent systematic review, investigating the efficacy and safety evaluation of autologous fat transplantation, platelet-rich plasma, and stromal vascular fraction in acne scars, found that 73% of cases showed excellent improvement, indicating the effectiveness of ADSVF (53). Similarly, two studies in our review indicated significant improvement in scar variables and scar sonographic measurements (26, 27).

Cao et al. studied various adipose tissue and fat derivatives in digital ulcer management in patients with systemic sclerosis. ADSVF showed the least change in terms of pain score and similar effects on wound healing to ADSCs and autologous fat (47). In our review, the ADSVF group showed significant improvement in the number of digital ulcers, Raynaud phenomenon, quality of life, and pain assessment. However, ADSVF was not compared to other adipose tissue and fat derivatives therapeutic modalities (30, 47).

Compared to ADSCs, ADSVF has an easy acquisition process and a variable cellular composition which contributes to better healing outcomes (23, 54, 55). However, limited expansion ability and wide variability of extraction methods are the primary challenges in clinical use of ADSVF (52).

### 4.4 Fat grafting

FG consists of mature adipocytes, preadipocytes, stem cells and multiple growth factors (11). The process of preparing FG includes, selecting a suitable donor site, typically the inner thighs and lower abdomen due to the high amount of viable adipocyte volume compared to other areas, followed by fat harvesting, processing and injection (56). While the primary mechanism of action of FG is volume enhancement, it also aids in tissue damage and improves the appearance of scars through various properties of adipocytes, stromal components and growth factors.

FG is less likely to cause an immunogenic response and is easier to process than ADSCs (57, 58). However isolated ADSCs or ADSVF have the regenerative potential of conventional FG without the need for large volume fat transfer (59).

Our review included four clinical trials across a wide variety of scar and wound types: ischemic digital ulcers in systemic sclerosis patients (20), chronic ulcers (arterial, venous, mixed arteriovenous, diabetogenic, and compressive types) (31), post-reduction mammoplasty scars, and superficial-deep burn wounds (22, 28). Patients with ischemic digital ulcers and burn wounds had better outcomes with FG treatment (20, 28), while patients with chronic ulcers and post-surgical scars had no significant improvement in wound healing parameters compared to control groups (22, 31). The broad range of wounds included in our review and the insufficient studies in literature for analysis make it difficult to draw conclusions on the effectiveness of FG in wound healing. These results were consistent with Malik et al. systematic review on the use of autologous fat grafting in the treatment of acute and chronic cutaneous wounds (59).

A recent systematic review investigated adipose-derived stem cells and their derivatives in burn treatment, was in line with our findings regarding the effectiveness of fat derivatives *in vitro*, but further clinical studies are needed (60). The addition of platelet-rich plasma (PRP) to adipose tissue and fat derivatives as therapeutic modalities is another emerging area of study in the field of regenerative medicine. PRP is used as an adjuvant to FG due to its role in increasing fat survival and potentiating its regenerative capabilities (61).

Our review found promising results regarding the use of PRP with FG for wound healing, especially concerning scar healing parameters and health-related quality of life (17, 21, 25). However, despite the positive outcomes, a final conclusion requires further studies sufficient for formal analysis. This is consistent with Smith et al. (62), which could not confirm PRP's benefit with FG for wound healing, due to the lack of standardized procedure protocols (62).

### 4.5 Status of current research and future recommendations

The positive outcomes reported with adipose tissue and fat derivatives in preclinical and clinical studies support a promising future for their use in regenerative medicine in general and wound healing specifically. However, concerns still exist regarding publication bias and underreporting of equivocal and negative results, an issue that has been raised before (59, 63). By examining multiple therapeutic modalities, including adipose tissue and fat derivatives, our review provides a holistic overview of adipose tissue's therapeutic potential, ensuring practitioners and researchers are well-informed about the extent of available strategies, their respective benefits, limitations, and gaps to address in future research. The development of standardized protocols for adipose tissue and fat derivative isolation, characterization, and application, as well as the endpoints investigated, is important to facilitate comparison across studies. Furthermore, large-scale randomized clinical trials comparing adipose tissue and fat derivatives to conventional treatments in skin wounds are needed, and trials aiming to compare the different adipose tissue modalities are encouraged. Additionally, investigating long-term safety profiles is crucial as well.

Our review showed that adipose-derived therapies illustrated benefits across different wound types. Nevertheless, the significant heterogeneity among the included studies; differences in adipose-derivatives types, wound types and underlying etiologies, chronicity, preparation techniques and delivery protocols, as well as outcomes assessment tools—limited our ability to conclude a direct head-to-head comparison between subgroups.

However, particular patterns are noted: ADSC-enriched therapies were promising in the treatment of chronic ischemic ulcers through their angiogenic and immunomodulatory mechanisms, while fat grafting combined with PRP appeared more beneficial in scar remodeling. Such observations can be regarded as hypothesis-generating for future high quality RCTs that will help provide effective subgroup comparisons. The development of standardized protocols for adipose tissue and fat derivative isolation, characterization, and application, as well as the endpoints investigated, is important to facilitate comparison across studies. Furthermore, large-scale randomized clinical trials comparing adipose tissue and fat derivatives to conventional treatments in skin wounds are needed, and trials aiming to compare the different adipose tissue modalities are encouraged. Additionally, investigating long-term safety profiles is crucial as well.

### 4.6 Limitations

Although our study is the first to our knowledge to present an overview of the current landscape of adipose tissue and fat derivative therapeutics in wound healing and scar management, a couple of challenges limit the generalizability of findings. The heterogeneity of the clinical trials in terms of procedure protocols, endpoints, and outcome measurements, poses a significant challenge to conducting a formal analysis. Definitive conclusions are not possible, as large-scale studies with high quality and low risk of bias are required. Furthermore, five included studies exhibited a high risk of bias, particularly in the domain of deviation from intended interventions and outcome measurement domain. Despite the fact that we provide comprehensive study-level extraction tables and supplementary tables, the extensive heterogeneity of studies included prevented us from summarizing findings in terms of a unified effect direction or pooled estimate. We believe that such aggregation would risk presenting specific results that would be misrepresenting and misleading.

Additionally, the exclusion of non-english studies may introduce language bias to our review, further systematic reviews should attempt to include multilingual literature to provide more comprehensive review. These methodological limitations may have introduced bias into the reported effects and should be considered when interpreting our findings.

### 4.7 Clinical applicability

The results of our review highlights two sides of adipose-derived therapies benefits; (1)Functional outcomes including;(pain reduction, wound healing and recurrence reduction), (2) Cosmetic and aesthetic outcomes like; (pigmentation reduction, and scar quality).Still, the clinical benefits differ based on wound type and intervention used. Preliminary evidence concluded from our review suggests that ADSVF-enriched interventions showed promise in chronic ulcers, while fat grafting—especially when combined with PRP was associated with better functional outcomes in scars treatment.

## 5 Conclusion

Adipose tissue and fat derived therapies significantly enhance wound healing, reduce pain, and improve cosmetic appearance, patient satisfaction, and health-related quality of life compared to conventional treatments. These therapies are effective across various wound types and scars, with minimal side effects. Given that many of the positive outcomes were reported in trials with high or unclear risk of bias, the current evidence should be considered preliminary. Further standardization and ongoing randomized trials are needed to consolidate the evidence and facilitate their integration into routine clinical practice.

## Data Availability

The original contributions presented in the study are included in the article/Supplementary Material, further inquiries can be directed to the corresponding author.

## Appendix 1

Complete search strategy

Inception—February 1, 2024

(“fat graft*” OR “fat transf*” OR “fat inject*” OR “fat derivate” OR “adipose graft*” OR “adipose stem cell*” OR “adipose derived stem cell*” OR “adipose tissue derivate*” OR “lipofill*” OR “lipotransf*” OR “lipomodell*” OR ASC* OR ADSC* OR “Conditioned Medium” OR “Medium, Conditioned” OR “Culture Medium, Conditioned” OR “Conditioned Culture Media” OR “Conditioned Media” OR “Media, Conditioned” OR “Conditioned Culture Medium” OR “Secretory Vesicle” OR “Vesicle, Secretory” OR “Secretory Granules” OR “Granule, Secretory” OR “Synaptic-Like Microvesicles” OR “Microvesicle, Synaptic-Like” OR “Synaptic-Like Microvesicle” OR SLMVs OR “Condensing Vacuoles” OR “Condensing Vacuole” OR “Vacuole, Condensing” OR “Zymogen Granules” OR “Granule, Zymogen” OR “Zymogen Granule” OR SVF OR nanofat OR “Stromal Vascular Fraction” OR “Total Stromal-Cells”) AND (“wound heal*” OR “wound management” OR “wound treat*” OR “ulcer heal*” OR “ulcer management” OR “ulcer treat*”).
