# Supplementary material for: Adipose tissue and fat-derived products in wound, ulcer, and scar management: a systematic review
Source: Front Surg. 2025 Oct 9;12:1666776. doi: 10.3389/fsurg.2025.1666776 (PMC12546055; doi:10.3389/fsurg.2025.1666776)
Supplement: Supplementary file 1 [file Table1.docx]

***Supplementary Material***

**Supplementary Table 1 : Summary of Studies Investigating Adipose-Derived Stem Cell-Conditioned Media in Wound, Ulcer, and Scar Management**

| **Author, Year** | **Wound/Ulcer/Scar** | **Intervention** | **Control** | **Outcomes** | | |
| --- | --- | --- | --- | --- | --- | --- |
|  |  |  |  | **Outcome Measurement Scales** | **Success: Wound Healing** | **Adverse Events** |
| **Zhou et al. 2013**[**(1)**](https://www.zotero.org/google-docs/?IW9PXe) | Ablative Fractional carbon dioxide laser resurfacing (FxCR) Scars | Allogeneic Adipose-Derived Stem Cells-Conditioned Media | Fetal bovine serum (FBS) free Dulbecco's modified Eagle's medium (DMEM) | The erythema index (EI).  The melanin index (MI).  Transepidermal water loss (TEWL).  R2, the primary parameter used to assess skin elasticity [(2)](https://www.zotero.org/google-docs/?sEimcR).  Histopathological examination. | **Less Erythema:** The intervention side showed a significant difference with lower EI than that of the other treated sites at day 1 post-treatment (P value < 0.05).  **Less Pigmentation:** The ADSC-CM-treated side presented statistically significantly lower average values of MI at days 1, 7, 14, and 21 (P value < 0.05).  **Comparative TEWL Changes:** The ADSC-CM-treated side showed a greater reduction in TEWL compared to the control side, although the difference was not statistically significant (P value > 0.05).  **Similar Histopathologic Analysis of Specimens:** No significant changes were observed in H&E staining and elastin staining. | Adverse events, including infection, prolonged erythema, and scarring, were not detected in either group participating in this study. |
| **Alinda et al. 2023**[**(3)**](https://www.zotero.org/google-docs/?AnYt3S) | Chronic Plantar Ulcers after Leprosy | Adipose mesenchymal stem cell-conditioned medium (every three days for eight weeks) | Framycetin gauze dressing(every three days for eight weeks) | The mean percentage of ulcer healing (ulcer size and depth).  Clinical pictures before and after application.  Immunohistochemistry and microscopic analysis. | **Full Healing (Complete Closure):** 10 patients (62.5%) in the intervention group versus 4 patients (25.0%) in the control group.  **Improved Healing (Ulcer Size Smaller at the End Compared with Baseline):** 6 patients (37.5%) in the ADSC-CM group versus 12 patients (75.0%) in the Framycetin group.  **Decrease in Ulcer Size:** A statistically significant reduction in ulcer mean size was observed in the intervention group starting from week 2 onwards (P value < 0.05).  **Reduction in Ulcer Depth:** The ADSC-CM-treated group had a greater decrease in ulcer depth compared to the control group (P value < 0.05).  **Better Vascularity Value:** Group 1 showed statistical differences in macrophages, fibroblasts, and vascularity value compared to Group 2 (P value < 0.05). | No adverse events or complications, including allergic contact dermatitis or infection, were observed in either group. |

[1. Zhou BR, Xu Y, Guo SL, Xu Y, Wang Y, Zhu F, et al. The effect of conditioned media of adipose-derived stem cells on wound healing after ablative fractional carbon dioxide laser resurfacing. BioMed Res Int. 2013;2013:519126.](https://www.zotero.org/google-docs/?X7AKWs)

[2. Akhtar N, Zaman SU, Khan BA, Amir MN, Ebrahimzadeh MA. Calendula extract: effects on mechanical parameters of human skin. Acta Pol Pharm. 2011;68(5):693–701.](https://www.zotero.org/google-docs/?X7AKWs)

[3. Alinda MD, Christopher PM, Listiawan MY, Endaryanto A, Suroto H, Rantam FA, et al. The efficacy of topical adipose mesenchymal stem cell-conditioned medium versus framycetin gauze dressing in chronic plantar ulcer of leprosy: A randomized controlled trial. Indian J Dermatol Venereol Leprol. 2023;89(5):656–64.](https://www.zotero.org/google-docs/?X7AKWs)
