# Supplementary material for: Adipose tissue and fat-derived products in wound, ulcer, and scar management: a systematic review
Source: Front Surg. 2025 Oct 9;12:1666776. doi: 10.3389/fsurg.2025.1666776 (PMC12546055; doi:10.3389/fsurg.2025.1666776)
Supplement: Supplementary file 2 [file Table2.docx]

***Supplementary Material***

**Supplementary Table 2 : Summary of Studies Investigating Adipose Derived Stromal Vascular Fraction in Wound, Ulcer, and Scar Management**

| **Author, Year** | **Wound/Ulcer/Scar** | **Intervention** | **Control** | **Outcomes** | | |
| --- | --- | --- | --- | --- | --- | --- |
|  |  |  |  | **Outcome Measurement Scales** | **Success: Wound Healing** | **Adverse Events** |
| **Chronic Ulcers** | | | | | | |
| **Zollino et al. 2019**[**(1)**](https://www.zotero.org/google-docs/?jrnoND) | Chronic leg ulcers | Centrifuged adipose tissue derived stromal vascular fraction | No experimental treatment was given to the control group | Numeric Pain Rating Scale (NRS)[(2)](https://www.zotero.org/google-docs/?dfp48S).  The Margolis Index (MI)[(3)](https://www.zotero.org/google-docs/?5jTEkc).  The wound healing process was assessed by observing the decrease in the initial area each week (square centimeters/week).  Histopathological examination. | **The healing rate at 24 weeks:** Six out of eight patients in the ADSVF group achieved healing, compared to four out of eight patients in the control group, with no significant difference observed (P = 0.30).  **Mean healing time (weeks):** There was a statistically significant difference between the ADSVF group and the control group (17.5 ± 7.6 and 24.5 ± 4.9, respectively) with a P-value of 0.036.  **The wound healing process (cm²/week):** Healing proceeded in the intervention group at 3.2 ± 8.0, which was not significantly different from the control group at 0.2 ± 0.4 (P = 0.37).  **The Margolis Index:** Fifty percent of patients in the intervention group achieved MI+, compared to 25% in the control group, with no statistically significant difference observed (P = 0.30).  **Drop in pain score:** The NRS decreased after the first week in the ADSVF group (2.7 ± 2.0), whereas in the comparative group, it was higher (6.6 ± 3.0) (P < 0.01). | One patient experienced perilesional dermatitis shortly after the ADSVF intervention, which resolved by the second week.[(4)](https://www.zotero.org/google-docs/?9Smko0) |
| **Tanios et al. 2021** | Chronic ulcers ( diabetic , venous , trophic and post-traumatic) | Adipose derived stromal vascular fraction | Conventional treatment (wound dressings) | Healing (rate and duration).  Photographs were taken to document the width and length of the ulcer before and after injection.  Clinical parameters of the ulcer.  Histopathological examination and scoring system[(5)](https://www.zotero.org/google-docs/?AqC0dM). | **The Healing Rate at 9 Weeks:** At 9 weeks, 46 patients in the ADSVF group achieved complete healing compared to 30 patients in the control group, with a statistically significant difference (P < 0.001).  **Shorter Healing Duration:** The intervention group had significantly shorter healing durations (7.87 ± 2.50 weeks compared to 13.87 ± 2.84 weeks in the control group; p = 0.000).  **Healing Method:** Forty-six patients in the ADSVF group healed using only ADSCs, while one patient required a split-thickness skin graft, and three required secondary sutures. | Three patients in the ADSVF group developed post-intervention infections, compared to 14 patients in the control group (P = 0.000). |
| **Post-Surgical and Traumatic Scars** | | | | | | |
| **Van Dongen et al. 2022**[**(6)**](https://www.zotero.org/google-docs/?8pEOIz) | Bilateral reduction mammoplasty scars | Tissue stromal vascular fraction of adipose tissue | Saline Injection ( Placebo) | Scar Appearance:  Assessed using the Patient and Observer Scar Assessment Scale (POSAS version 2.0)[(7)](https://www.zotero.org/google-docs/?7m18BG).  Photographic Evaluation of Scar Appearance.  Histological Examination and Skin Biopsy. | **Postoperative Scar Appearance at the 6-Month Follow-Up:** The injection of tSVF significantly improved postoperative scar appearance at the 6-month follow-up. The mean total score of the patient part in the POSAS questionnaire was 21 ± 15.0 in the tSVF group and 24.5 ± 13.0 in the placebo group (p < 0.05). Similarly, the mean total score of the observer part in the POSAS questionnaire was 18.8 ± 11.3 in the tSVF group and 23.6 ± 11.2 in the placebo group (p < 0.01).  **Postoperative Scar Appearance at the 12-Month Follow-Up:** The injection of tSVF did not improve postoperative scar appearance at the 12-month follow-up. The average total score of the patient part in the POSAS questionnaire was 14.4 ± 7.6 in the tSVF group versus 15.3 ± 9.0 in the control group (p > 0.05). Likewise, the average total score of the observer part in the POSAS questionnaire was 14.5 ± 6.4 in the tSVF group compared to 14.6 ± 8.8 in the control group (p > 0.05).  **Postoperative Administration of tSVF Did Not Enhance Collagen Architecture.** | Not Reported |
| **Kwon et al. 2023**[**(8)**](https://www.zotero.org/google-docs/?UJ7Tse) | Traumatic and surgical scars | Stromal vascular fraction injection | Normal Saline Injection ( Placebo) | Scar appearance assessed using the Patient and Observer Scar Assessment Scale (POSAS)[(9)](https://www.zotero.org/google-docs/?TZvbeu). | **Postoperative Scar Appearance at the 6-Month Follow-Up:** The injection of SVF significantly improved postoperative scar appearance at the 6-month follow-up. The mean total score of the patient part in the POSAS questionnaire was 13.13 ± 2.70 in the SVF group and 18.56 ± 3.29 in the placebo group (p < 0.001). Similarly, the mean total score of the observer part in the POSAS questionnaire was 15.88 ± 2.58 in the SVF group and 22.75 ± 4.22 in the placebo group (p < 0.01)**.** | Except for two patients who reported mild pain within two days post-surgery, no other patients experienced adverse effects during the six-month monitoring period. |
| **Acne Scars** | | | | | | |
| **Roohaninasab et al. 2022**[**(10)**](https://www.zotero.org/google-docs/?S6jGgO) | Acne scars | Subcision technique with stromal vascular fraction injection | Subcision technique only | Recovery Rate: Assessed using a mental scoring system based on patient and doctor assessments, along with visual evaluation of scars.  Biometric Evaluation of Patients at 3 Months: This included assessment of neocollagenesis levels using a Skin Ultrasound Imaging System and the VisioFace technique. | **Data Obtained from VisioFace (Including Volume, Area, and Depth):**  The combined therapy involving SVF and subcision resulted in a significant improvement compared to the subcision method alone, particularly in terms of volume and area (P < 0.001). The reduction in scar depth was greater in the SVF group; however, this decrease was not statistically significant (P = 0.438).  **Ultrasound Imaging System Data:**  A significant increase in complete, epidermal, and dermal thickness, as well as epidermal density variables, was noted between the control and case groups (P < 0.05).  **Patient/Doctor Satisfaction:**  For patients, the average satisfaction score was 7.10 ± 0.99 in the SVF group compared to 5.30 ± 1.25 in the control group (P = 0.003).  For doctors, the mean satisfaction score was 7.10 ± 0.74 in the SVF group versus 5.50 ± 0.53 in the control group (P = 0.004). | No adverse reactions, including bleeding and infection at the sites of fat removal and subcision, were detected in any of the patients. |
| **Behrangi et al. 2022**[**(11)**](https://www.zotero.org/google-docs/?Br2PJw) | Acne scars | Combination of nanofat subcutaneously and Stromal vascular Fraction intradermally | Nanofat subcutaneously | Scar Variables (Volume, Area, and Depth): Assessed by digital reimaging after 1 month.  Assessment of Neocollagenesis Levels Using a Skin Ultrasound Imaging System. | **Scar Variables (Volume, Area, and Depth):** Significantly decreased after 1 month in the SVF group compared to the control (P value < 0.05).  **Volume:** 84.23 ± 44.41 in the SVF group compared to 99.86 ± 40.97 in the control group.  **Area:** 8.43 ± 3.87 in the SVF group versus 9.36 ± 3.69 in the control group.  **Depth:** 9.14 ± 0.90 in the SVF group compared to 10.14 ± 0.90 in the control group.  **Ultrasound Imaging System Data**: Faster improvement was observed in the SVF group with no significant difference in sonographic variables between the case and control groups (P > 0.05). | Not Reported |
| **Systemic Sclerosis Ulcers** | | | | | | |
| **Iglesias et al. 2023**[**(12)**](https://www.zotero.org/google-docs/?VXAbg9) | Systemic sclerosis wounds (digital ulcers) | Medical treatment and Local adipose derived stromal vascular fraction mixed with micrografts | Medical treatment only ( stable vasoactive and immunosuppressive therapies) | Pain: Assessed using a numeric scale ranging from 1 to 10.  Quality of Life: Assessed using the Short Form 36 questionnaire.  Digital Oximetry: Assessed using Transcutaneous Oximetry (%).  Raynaud Phenomenon:  Frequency: Number of events per day/week.  Duration: Minutes in each event.  Intensity: Color classification (white, purple, and red) of events.  Digital Ulcers: Assessed by counting the number of ulcers.  Digital Total Active Motion: Assessed using Digital Manual Goniometry.  Thumb Opposition: Assessed using the Kapandji Test.  Nail Capillaroscopic Patterns: Assessed using Nailfold Videocapillaroscopy (early, active, and late patterns).  Skin Affection of the Hand: Assessed using the Modified Rodnan Skin Score (mRSS).  Hand Function Assessment.  Health Status Evaluation. | **Digital Oximetry (SpO2), Digital Total Active Motion, Thumb Opposition, Hand Function, Health Status, and Disability Index:** No significant differences were detected between the experimental and control groups.  **Pain Assessment:**  ***Initial Days Post-Intervention:*** In the ADSVF group, the median pain score gradually increased from 4.6 to 7.5, then steadily declined, returning to baseline levels by day 21, and continued to decrease thereafter (P < 0.01). In contrast, pain decreased in the control group, although insignificantly.  ***168 Days Follow-Up:*** Pain showed a significant improvement in the experimental group compared to the control (P < 0.05).  **Quality of Life Assessment:** Significant improvements were reported in the ADSVF group (P < 0.05) at the end of the study.  **Raynaud Phenomenon:** Both groups demonstrated improvements in frequency, duration, and intensity. However, no significant differences were observed between the experimental and control groups when comparing them (P > 0.05).  **Skin Affection of the Hand:** Showed a significant improvement in the control group (P < 0.05).  **Number of Digital Ulcers:** Decreased significantly in the experimental group by the end of the study (P < 0.01).  **Capillaroscopic Patterns:** Both groups exhibited late patterns with no significant change in the number of nailfold capillary loops between the groups.  ***ADSVF Group:*** One patient exhibited an improved capillaroscopic pattern changing from late to early.  ***Control Group:*** One patient exhibited a worsened capillaroscopic pattern changing from early to late. | No adverse events were observed in either group. Ulcer recurrence was noted in one patient in the ADSVF group compared to three patients in the control group. |

[1. Zollino I, Campioni D, Sibilla MG, Tessari M, Malagoni AM, Zamboni P. A phase II randomized clinical trial for the treatment of recalcitrant chronic leg ulcers using centrifuged adipose tissue containing progenitor cells. Cytotherapy. 2019 Feb;21(2):200–11.](https://www.zotero.org/google-docs/?DEyr0w)

[2. Ruan X, Padnos IW, Kaye AD. Validation of a New “Objective Pain Score” vs. “Numeric Rating Scale” For the Evaluation of Acute Pain: A Comparative Study. Anesthesiol Pain Med. 2016 Jun 6;6(4):e38886.](https://www.zotero.org/google-docs/?DEyr0w)

[3. Kantor J, Margolis DJ. A multicentre study of percentage change in venous leg ulcer area as a prognostic index of healing at 24 weeks. Br J Dermatol. 2000 May;142(5):960–4.](https://www.zotero.org/google-docs/?DEyr0w)

[4. Tanios E, Ahmed TM, Shafik EA, Sherif MF, Sayed D, Gaber N, et al. Efficacy of adipose-derived stromal vascular fraction cells in the management of chronic ulcers: a randomized clinical trial. Regen Med. 2021 Nov;16(11):975–88.](https://www.zotero.org/google-docs/?DEyr0w)

[5. Hazrati M, Mehrabani D, Japoni A, Montasery H, Azarpira N, Hamidian-shirazi AR, et al. Effect of Honey on Healing of Pseudomonas aeruginosa Infected Burn Wounds in Rat. J Appl Anim Res. 2010 Jun 1;37(2):161–5.](https://www.zotero.org/google-docs/?DEyr0w)

[6. van Dongen JA, van Boxtel J, Uguten M, Brouwer LA, Vermeulen KM, Melenhorst WB, et al. Tissue Stromal Vascular Fraction Improves Early Scar Healing: A Prospective Randomized Multicenter Clinical Trial. Aesthet Surg J. 2022 Jun 20;42(7):NP477–88.](https://www.zotero.org/google-docs/?DEyr0w)

[7. van de Kar AL, Corion LUM, Smeulders MJC, Draaijers LJ, van der Horst CMAM, van Zuijlen PPM. Reliable and feasible evaluation of linear scars by the Patient and Observer Scar Assessment Scale. Plast Reconstr Surg. 2005 Aug;116(2):514–22.](https://www.zotero.org/google-docs/?DEyr0w)

[8. Kwon H, Lee S, Kim J, Song SH. Efficacy and safety of stromal vascular fraction on scar revision surgery: a prospective study. J Dermatol Treat. 2023 Jan 19;34(1):2171260.](https://www.zotero.org/google-docs/?DEyr0w)

[9. Draaijers LJ, Tempelman FRH, Botman YAM, Tuinebreijer WE, Middelkoop E, Kreis RW, et al. The patient and observer scar assessment scale: a reliable and feasible tool for scar evaluation. Plast Reconstr Surg. 2004 Jun;113(7):1960–5; discussion 1966-1967.](https://www.zotero.org/google-docs/?DEyr0w)

[10. Roohaninasab M, Seifadini A, Atefi N, Sadeghzadeh-Bazargan A, Goodarzi A, Hanifnia AR, et al. Evaluating the effectiveness of stromal-vascular fraction (SVF) cells along with subcision method in the treatment of acne scars: A double-blind randomized controlled clinical trial study. J Cosmet Dermatol. 2022 Dec;21(12):6928–38.](https://www.zotero.org/google-docs/?DEyr0w)

[11. Behrangi E, Moradi S, Ghassemi M, Goodarzi A, Hanifnia A, Zare S, et al. The investigation of the efficacy and safety of stromal vascular fraction in the treatment of nanofat-treated acne scar: a randomized blinded controlled clinical trial. Stem Cell Res Ther. 2022 Jul 15;13(1):298.](https://www.zotero.org/google-docs/?DEyr0w)

[12. Iglesias M, Torre-Villalvazo I, Butrón-Gandarillas P, Rodríguez-Reyna TS, Torre-Anaya EA, Guevara-Cruz M, et al. Adipose derived stromal vascular fraction and fat graft for treating the hands of patients with systemic sclerosis. A randomized clinical trial. PloS One. 2023;18(8):e0289594.](https://www.zotero.org/google-docs/?DEyr0w)
