# Supplementary material for: Adipose tissue and fat-derived products in wound, ulcer, and scar management: a systematic review
Source: Front Surg. 2025 Oct 9;12:1666776. doi: 10.3389/fsurg.2025.1666776 (PMC12546055; doi:10.3389/fsurg.2025.1666776)
Supplement: Supplementary file 3 [file Table3.docx]

***Supplementary Material***

**Supplementary Table 3 : Summary of Studies Investigating Fat Grafting in Wound, Ulcer, and Scar Management**

| **Author, Year** | **Wound/Ulcer/Scar** | **Intervention** | **Control** | **Outcomes** | | |
| --- | --- | --- | --- | --- | --- | --- |
|  |  |  |  | **Outcome Measurement Scales** | **Success: Wound Healing** | **Adverse Events** |
| **Fat Grafting With Platelet Rich Plasma (FG+PRP)** | | | | | | |
| **Cervelli et al. 2012**[**(1)**](https://www.zotero.org/google-docs/?fjsAli) | Traumatic Scars | **-Group A** ( fat grafts mixed with PRP during months 1 and 3).  **-Group C** (Graft ⁄ PRP  treatment, with the laser therapy during  months 1 and 3 delivered 7 days after the graft ⁄ PRP  treatment). | **-Group B** ( four sessions of laser treatment with the 1540  nm non ablative laser (one per month)). | The cosmetic outcome of the scar, including its color, texture, shape, surface appearance (whether it looks matte or shiny), size, and overall appearance, assessed using the Manchester Scar Scale (MSS) [(2)](https://www.zotero.org/google-docs/?nlDugM).  Patient satisfaction is assessed at a 6-month appointment using a structured questionnaire to grade the scar's aesthetic and functional quality, rated by the patient as excellent, good, fair, or poor. | **The cosmetic outcome of the scar:**  PRP significantly enhanced the effectiveness of scar treatments.  The most effective scar treatment was the combination of fat/PRP and nonablative laser resurfacing in group C.  Group C demonstrated increases in wound healing of 22% and 11% compared to groups A and B, respectively.  **Patient satisfaction:**  The majority of patients were satisfied with the treatment, with all patients reporting improvement in scar texture.  At 6 months post-treatment, 84% of patients evaluated the scar appearance as showing good to excellent improvement, while 16% rated the appearance as poor to fair. | **Mild side-effects reported included:**  Transient erythema lasting 2–3 days.  Edema lasting 1–2 days.  Temporary hyperpigmentation reported in 4 patients. |
| **Smith et al. 2020**[**(3)**](https://www.zotero.org/google-docs/?I6FmNo) | Diabetic foot ulcers  ( Two papers reporting the results of the same clinical trial) | **-Group 1** (fat grafting)  **-Group 2** ( fat grafting with PRP ) | **-Group 3** (podiatry standard of care) | Wound Size and Healing: Assessed by 3D volumetric wound photographs using an Eykona wound management system camera.  Pressure Ulcer Scale for Healing (PUSH Score)[(4)](https://www.zotero.org/google-docs/?0QyetZ).  Cost of Dressings.  Health-Related Quality of Life: Assessed using a validated health-related quality of life (HRQoL) questionnaire specific to diabetic foot ulcer patients [(5)](https://www.zotero.org/google-docs/?5eCaQA).  Histological and Immunological Examination. | **Wound Size:** No significant difference in change in wound volume or area over 12 weeks was reported among any of the groups (P > 0.05).  **Wound Healing:** No significant difference in time to healing was observed between the intervention and control groups (P > 0.05). Complete healing was reported by:  Two patients from the (FG+PRP) group (at 73 and 80 days).  Two patients from the Fat group (at 78 and 80 days).  One patient from the control group (at 64 days).  **PUSH Score:** No significant difference was found in the change in PUSH score over 12 weeks among the groups (P > 0.05).  **Health-Related Quality of Life:**  Post-trial, significant differences between groups were observed in various domains of Health-Related Quality of Life (HRQoL). The control group consistently showed lower scores compared to the Fat and (FG+PRP) groups, particularly in areas such as daily activities, emotions, compliance, friends, treatment, and financial aspects. |  |
| **Nolan et al. 2022**[**(6)**](https://www.zotero.org/google-docs/?V3iOr2) |  |  |  |  |  | Three cases of infection occurred in the target ulcers, all of which resolved with oral antibiotics.  One patient experienced an allergic reaction to prescribed antibiotics.  One patient developed a superficial fluid collection along the cannula track from fat grafting, requiring drainage.  Four serious adverse events resulted in hospital admission, including two for infection in non-trial ulcers requiring debridement, one for chest sepsis, and one for heart failure.  During the trial period, four patients developed separate diabetic foot ulcers, and one patient developed a necrotic toe. |
| **Fat Grafting Without Platelet Rich Plasma** | | | | | | |
| **Del Papa et al. 2019**[**(7)**](https://www.zotero.org/google-docs/?Ggonmk) | Digital Ischemic ulcers (IDU) in patients with systemic sclerosis | Fat grafting with medical treatment | Sham Procedure ( false liposuction and local injection of saline solution) with medical treatment | IDU Healing and Healing Time  Pain Score: Measured by Visual Analog Scale (VAS)  Nailfold Videocapillaroscopy (NVC) | **IDU Healing:**  IDU healing was observed in 23 out of 25 patients treated with fat grafting (FG), compared to 1 out of 13 patients in the control group (P < 0.0001).  Patients in the experimental group had significantly longer-lasting IDUs compared to patients in the control arm (P < 0.01).  **Pain Improvement:**  Significant reduction in pain intensity was recorded after 4 and 8 weeks in the fat grafting (FG) group (P < 0.0001).  **Capillary Numbers:**  There was a significant increase in capillary numbers in the affected finger after 4 and 8 weeks in the intervention group (P < 0.0001). | No adverse events were observed in either group. |
| **Kemaloğlu et al. 2021**[**(8)**](https://www.zotero.org/google-docs/?IOIWzd) | Post-Reduction mammoplasty scars | -Group 1 ( Fat Graft)  -Group 2 ( Nanofat-enriched fat graft) | -Group 3 (No additional treatment was applied to the surgical incisions ) | Vancouver Scar Scale (VSS): Assessed by three independent blinded reviewers at the postoperative six-month follow-up.  Visual Analogue Scale (VAS): Assessed by patients on a scale of 1 to 10 (1 indicating best results and 10 indicating worst results) at the postoperative six-month follow-up. | **VSS Score:**  Comparing the three groups, a significant reduction was observed in both experimental groups compared to the control group in all variables except for scar height.  In the two intervention groups, pigmentation scores were significantly lower in the Nanofat group compared to the fat group (p = 0.005).  No significant differences were observed between the two experimental groups in terms of vascularization, pliability, and scar height scores (p = 0.084, p = 0.988, and p = 0.980, respectively).  **VAS Scores:**  Comparing the three groups, significantly lower values were observed in both the fat and Nanofat groups compared to the control group (p = 0.001 for both groups).  However, no statistical difference in VAS scores was detected between the fat and Nanofat groups (p = 0.060) within the experimental groups. | In the control group, one patient developed nipple necrosis. |
| **Abouzaid et al. 2022**[**(9)**](https://www.zotero.org/google-docs/?BzRKFP) | Superficial and deep dermal burn wounds | Single injection of autologous fat grafting and dressing with nanofat | Conventional methods with serial dressing and use of topical agents e.g., Silver Sulfadiazine, mafenide and/ or  others | The Wong-Baker FACES pain rating scale was used to assess pain in both groups.  Histopathological examination was conducted. | **Healing Time:** The Fat/Nanofat group showed faster complete healing compared to the control group.  **Total Hospital Stay, Visits to the Operating Room, and Outpatient Clinic Visits Post-Discharge:** A significant reduction in total hospital stay, frequency of visits to the operating room, and outpatient clinic visits post-discharge was observed in the Fat/Nanofat group compared to the control group (p < 0.001).  **Hospital Readmission:**  Fat/Nanofat group: 20% of cases.  Control group: 38% of cases.  **Need for Further Split-Thickness Skin Grafting:** A significant reduction in the need for further skin grafting was observed in the intervention group (p = 0.003).  Fat/Nanofat group: 20% of cases.  Control group: 48% of cases.  **Contracture Formation:** The incidence of contracture formation decreased significantly in the experimental group (p < 0.002).  Fat/Nanofat group: 10% of cases.  Control group: 36% of cases.  **Scar Texture:** Improvement in texture was observed in the Fat/Nanofat group (p < 0.001).  Fat/Nanofat group: 80% of cases had a smooth texture.  Control group: 32% of cases had a smooth texture.  Hypertrophic or Keloid Scars:  Fat/Nanofat group: 10% of cases.  Control group: 72% of cases.  **Pain Assessment:** The experimental group exhibited lower pain scores compared to the control group. | Not Reported |
| **Thamm et al. 2023**[**(10)**](https://www.zotero.org/google-docs/?e6y66W) | Chronic Leg Ulcers | Sublesional fat graft Injection | Saline solution (0.9% NaCl) injection | Visual Analog Scale (VAS) was used to assess the level of pain intensity.  Wound size was assessed using photographs taken at different intervals.  Histological and immunological examinations were conducted. | **Wound Size:** The difference between the intervention and control groups was not statistically significant (p = 0.081).  Intervention group: Mean final wound size on Day 60 was 45.4 ± 34.6%.  Control group: Mean final wound size on Day 60 was 69.3 ± 46.7%.  **Pain Levels:** The largest disparity in pain levels between groups occurred during the immediate postoperative period, spanning from Day 3 to Day 7. However, these differences did not reach statistical significance (Day 3, p = 0.363; Day 7, p = 0.051).  **Qualitative Tissue Reactions:** No qualitative differences were found between the Fat and control groups.  **Neovascularization:** The mean degree of neovascularization was consistently higher in the experimental group at all assessment times throughout the study. However, these results did not show statistical significance (p > 0.05). | Three patients showed clinical signs of a local wound infection during the study. |

[1. Cervelli V, Nicoli F, Spallone D, Verardi S, Sorge R, Nicoli M, et al. Treatment of traumatic scars using fat grafts mixed with platelet-rich plasma, and resurfacing of skin with the 1540 nm nonablative laser. Clin Exp Dermatol. 2012 Jan;37(1):55–61.](https://www.zotero.org/google-docs/?bBAC6p)

[2. Beausang E, Floyd H, Dunn KW, Orton CI, Ferguson MW. A new quantitative scale for clinical scar assessment. Plast Reconstr Surg. 1998 Nov;102(6):1954–61.](https://www.zotero.org/google-docs/?bBAC6p)

[3. Smith OJ, Leigh R, Kanapathy M, Macneal P, Jell G, Hachach-Haram N, et al. Fat grafting and platelet-rich plasma for the treatment of diabetic foot ulcers: A feasibility-randomised controlled trial. Int Wound J. 2020 Dec;17(6):1578–94.](https://www.zotero.org/google-docs/?bBAC6p)

[4. de Gouveia Santos VLC, Sellmer D, Massulo MME. Inter rater reliability of Pressure Ulcer Scale for Healing (PUSH) in patients with chronic leg ulcers. Rev Lat Am Enfermagem. 2007;15(3):391–6.](https://www.zotero.org/google-docs/?bBAC6p)

[5. Abetz L, Sutton M, Brady L, McNulty P, Gagnon DD. The Diabetic Foot Ulcer Scale (DFS): a quality of life instrument for use in clinical trials. Pract Diabetes Int. 2002;19(6):167–75.](https://www.zotero.org/google-docs/?bBAC6p)

[6. Nolan GS, Smith OJ, Heavey S, Jell G, Mosahebi A. Histological analysis of fat grafting with platelet-rich plasma for diabetic foot ulcers-A randomised controlled trial. Int Wound J. 2022 Feb;19(2):389–98.](https://www.zotero.org/google-docs/?bBAC6p)

[7. Del Papa N, Di Luca G, Andracco R, Zaccara E, Maglione W, Pignataro F, et al. Regional grafting of autologous adipose tissue is effective in inducing prompt healing of indolent digital ulcers in patients with systemic sclerosis: results of a monocentric randomized controlled study. Arthritis Res Ther. 2019 Jan 7;21(1):7.](https://www.zotero.org/google-docs/?bBAC6p)

[8. Kemaloğlu CA, Özyazgan İ, Gönen ZB. Immediate fat and nanofat-enriched fat grafting in breast reduction for scar management. J Plast Surg Hand Surg. 2021 Jun;55(3):173–80.](https://www.zotero.org/google-docs/?bBAC6p)

[9. Abouzaid AM, El Mokadem ME, Aboubakr AK, Kassem MA, Al Shora AK, Solaiman A. Effect of autologous fat transfer in acute burn wound management: A randomized controlled study. Burns J Int Soc Burn Inj. 2022 Sep;48(6):1368–85.](https://www.zotero.org/google-docs/?bBAC6p)

[10. Thamm OC, Eschborn J, Zimmermann L, Dekker C, Martin H, Brockmann M, et al. Sublesional fat grafting leads to a temporary improvement of wound healing in chronic leg ulcers: A prospective, randomised clinical trial. Wound Repair Regen Off Publ Wound Heal Soc Eur Tissue Repair Soc. 2023;31(5):663–70.](https://www.zotero.org/google-docs/?bBAC6p)
